# Insights Into Hepatitis B Awareness and Vaccination Trends Among Healthcare Providers: A Comprehensive Study

**DOI:** 10.7759/cureus.69067

**Published:** 2024-09-10

**Authors:** Lavanya Balaji, Tasneem Banu, Abiramasundari VK, Neelusree Prabhakaran

**Affiliations:** 1 Department of Microbiology, Saveetha Medical College and Hospital, Saveetha Institute of Medical and Technical Sciences, Saveetha University, Chennai, IND

**Keywords:** healthcare professional, hepatitis b infection, hepatitis b vaccine, infection prevention and control, knowledge attitudes and practices

## Abstract

Background: Hepatitis B virus (HBV) infection poses significant occupational risks to healthcare workers (HCWs) worldwide. Understanding the knowledge, attitudes, and practices (KAPs) of HCWs regarding HBV infection and vaccination is crucial for developing effective preventive strategies. This study aims to assess the KAPs of the HCWs regarding HBV transmission, prevention, and vaccination in Saveetha Medical College and Hospital, Thandalam, Tamil Nadu.

Materials and methods: A cross-sectional analytical study was conducted at Saveetha Medical College and Hospital from January 2024 to May 2024. Participants included doctors, interns, nurses, and technicians (n = 112) who completed a validated questionnaire assessing their KAPs regarding HBV infection, prevention, and vaccination. The data were analyzed using the SPSS version 24.0 software (IBM Corp., Armonk, NY). The categorical data were presented in frequencies and percentages. The statistical significance was analyzed using the Kruskal-Wallis test to determine their statistical significance (p < 0.05).

Results: The majority of respondents demonstrated good knowledge (mean score = 6.40), positive attitudes (mean score = 7.29), and appropriate practices (mean score = 7.11) toward HBV prevention and vaccination. Significant differences were observed based on designation with p < 0.05 (p = 0.04), with doctors consistently exhibiting higher KAP scores (mean score = 8.7) compared to nurses (mean score = 6.24) and technicians (mean score = 7.36).

Conclusion: Our study found that while most HCWs understand hepatitis B and support vaccination, doctors exhibit superior knowledge compared to nurses and technicians. High adherence to prevention protocols is noted, but targeted educational interventions, such as workshops and continuous medical education, are needed to address knowledge gaps. Regular updates and mentorship programs can enhance understanding and foster a collaborative environment, leading to more effective hepatitis B prevention and improved patient care.

## Introduction

Hepatitis B virus (HBV) infection is a significant global health issue, affecting around two billion people worldwide and causing about one million deaths annually [1]. Of these cases, 350 million individuals develop chronic infections and become long-term carriers of the virus [2]. Healthcare workers (HCWs) are at a higher risk of HBV exposure due to their frequent contact with blood and bodily fluids. Most of these exposures (75%) occur through contact with the skin, posing a higher risk of HBV transmission than exposure through mucous membranes or the skin [3,4]. Despite available preventive measures such as vaccination and postexposure prophylaxis (PEP), HBV remains a major hazard for HCWs. The World Health Organization (WHO) points out that the rate of occupational injuries among HCWs varies globally, ranging from 0.2 to 4.7 injuries annually [5,6]. In Asia, where the prevalence of HBV is estimated to be between 15% and 21% [7], HCWs are particularly vulnerable to infection. Studies show that 14.4% of hospital workers are infected with HBV, and 1.4% are infected with hepatitis C virus (HCV) [8]. The risk of HCWs developing clinical hepatitis after sustaining injuries from needles contaminated with HBV is reported to range from 1%-6% to 22%-31% in South India [9]. Current preventive strategies include vaccination and, when needed, PEP. The Centers for Disease Control and Prevention (CDC) and WHO both recommend universal HBV vaccination for all HCWs, consisting of three doses administered over a six-month period, with follow-up testing for hepatitis B surface antibody (anti-HB) levels to ensure sufficient immunity [10]. It is important to note that approximately 5%-10% of immunocompetent individuals do not develop sufficient immunity following the standard vaccination schedule [11].

Vaccination of HCWs is vital in preventing the spread of HBV. According to CDC guidelines, infected HCWs should refrain from performing exposure-prone procedures if their HBV DNA levels exceed 1,000 international units/mL, and their treatment is monitored by an expert panel [12]. Following standard practices and using proper barrier precautions are essential to prevent HBV transmission when dealing with patients in healthcare settings. Pre-exposure vaccines are also recommended. In cases of exposure to blood or bodily fluids, PEP may include passive immunization with hepatitis B immunoglobulin and vaccination with the hepatitis B vaccine. However, the most cost-effective way to prevent and manage hepatitis B is through pre-exposure vaccination and adhering to standard precautions [13]. Despite numerous publications on programs and strategies to prevent transmission, HBV and HCV infections continue to be significant public health concerns.

The effectiveness of preventive measures depends on the knowledge, attitudes, and practices (KAPs) of HCWs regarding HBV. Studies have indicated that HCWs' behaviors and adherence to preventive protocols are influenced by their understanding and attitudes toward HBV infection [14]. However, there is a significant gap in research on the KAPs of HCWs in South India regarding HBV infection transmission, prevention, and vaccination.

While extensive literature exists on HBV prevention strategies and their effectiveness, limited research has focused on the KAPs of HCWs in South India concerning HBV. Understanding HCWs' awareness, attitudes, and practices is crucial in identifying knowledge gaps and compliance issues with preventive measures. Such insights are necessary to develop training and intervention strategies to improve HBV prevention and control in hospital settings. However, there is a scarcity of studies focusing on the KAP level of HCWs in South India about HBV infection. In public health research, KAP surveys are widely employed to gather comprehensive information about what participants know, believe, and do regarding a specific topic. In this context, knowledge pertains to the depth of understanding, attitude reflects feelings and intentions, and practice encompasses how knowledge and attitude are put into action [15].

This study aims to assess the existing knowledge gap by examining the KAPs of doctors, nurses, technicians, and interns regarding HBV infection transmission, prevention, and vaccination in a tertiary care center in South India. By identifying knowledge deficiencies, negative attitudes, and inadequate practices, the study aims to inform the development of targeted strategies to improve HBV prevention and control efforts. This research is crucial for enhancing the safety of HCWs and reducing the incidence of HBV infection in healthcare settings.

## Materials and methods

Study design and study setting

A cross-sectional analytical study was carried out at Saveetha Medical College and Hospital in Thandalam, Tamil Nadu, to evaluate the KAPs of doctors, interns, nurses, and technicians regarding hepatitis B infection, prevention, and vaccination. The study involved 112 participants and was conducted from October 2023 to January 2024. The analysis was completed in March 2024. Ethical clearance was obtained from the hospital's institutional review board before conducting the study (262/07/2023/PG/SRB/SMCH). The participants in the referenced questionnaire provided consent in accordance with ethical standards and procedures.

Inclusion criteria

Participants must be actively employed as HCWs, including doctors, nurses, technicians, and interns, in our hospital.

Exclusion criteria

HCWs with less than one year of experience in a hospital setting were excluded from the study. Participants who had incomplete or missing responses on the KAP assessment were also excluded. To avoid response bias, individuals who had participated in similar studies or surveys on hepatitis B within the past year were excluded from the study.

Data collection and questionnaire development

Upon receiving informed consent, the study participants were asked to complete a validated questionnaire with 12 questions focusing on hepatitis B transmission, prevention, and vaccination. The questionnaire was developed based on an extensive review of relevant literature, including peer-reviewed articles, guidelines from health organizations like the WHO and the CDC, and previous studies on hepatitis B and HCWs. The literature review aimed to ensure that the questionnaire covered all crucial aspects of hepatitis B transmission, prevention, and vaccination, identifying gaps in knowledge, attitudes toward vaccination, and practices in clinical settings. It also helped to select validated questions from similar studies that could be adapted to this research. The questionnaire was designed to cover essential aspects of hepatitis B prevention and vaccination, such as basic knowledge of hepatitis B transmission, the importance of vaccination, attitudes toward vaccination, and common preventive practices in healthcare environments. It was divided into four sections: demographics, knowledge, attitudes, and practices, each carefully crafted to meet specific objectives. To ensure the questionnaire's clarity and effectiveness, a pilot test was undertaken with a small group of HCWs from the same tertiary care center representative of the study’s target population. Feedback from the pilot test was used to refine the questions and ensure the questionnaire's reliability and validity. The final version of the questionnaire was distributed via Google Forms to doctors, interns, nurses, and lab technicians, allowing for an efficient and accessible process. Respondents used a 3-point Likert scale to provide nuanced and easily analyzable responses, covering their knowledge of hepatitis B, attitudes toward vaccination, perceptions regarding the disease, preferred preventive measures, and suggestions for improving vaccination practices. The validated questionnaire is provided in Appendix 1.

Data management and scoring system for the questionnaire

The data were gathered using Google Forms and Microsoft Excel (Microsoft, Redmond, WA). The questionnaire uses a 3-point Likert scale with responses of yes (2 points), maybe (1 point), and no (0 point). Respondents are scored based on their answers, with scores tallied for each section. Cumulative scores of 6 or higher indicate good knowledge, a positive attitude, and good practice. In contrast, cumulative scores under 6 indicate poor knowledge, an unfavorable attitude, and poor compliance with recommended practices. Integrated scores from all three sections provide a comprehensive evaluation of hepatitis B-related KAP. The scores of the respondents are provided in Appendix 2.

Statistical analysis

Statistical analysis involved calculating quantitative data's mean and standard deviation, while categorical variables were summarized using frequencies and percentages. The relationships between sociodemographic factors and respondents' KAP concerning hepatitis B infection, prevention, and vaccination were assessed. Data were presented in tables and graphs, with participants' anonymity preserved. The Kruskal-Wallis test assessed statistical significance, with a threshold of p < 0.05 denoting significance. Correlation and multivariate regression analyses were conducted to explore associations between demographic factors and KAP and the relationship between KAP and other variables. All analyses were performed using IBM SPSS Statistics version 24 for Windows (IBM Corp., Armonk, NY).

## Results

Demographic characteristics of the respondents

**Figure 1:**
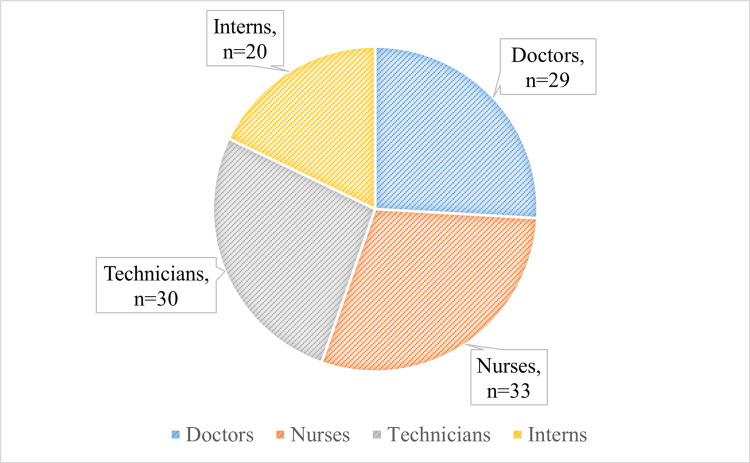
Distribution of respondents as per their designation The data are presented as n

**Figure 2:**
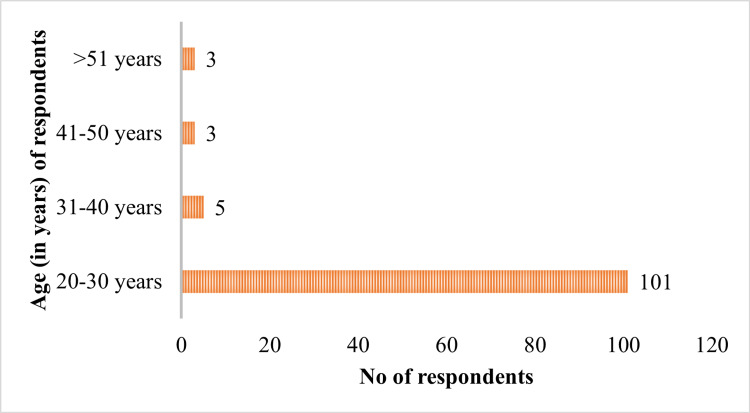
Agewise distribution of respondents The data are presented as n

**Figure 3:**
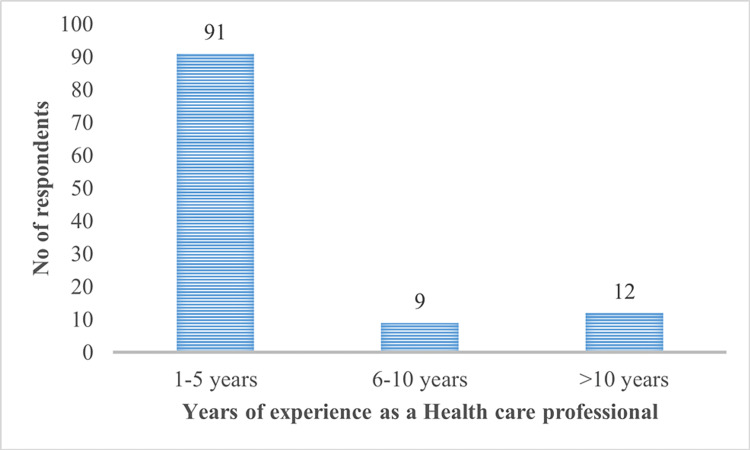
Distribution of respondents by years of experience as a healthcare professional The data are presented as n

The professional breakdown of the participants included nurses (29.5%, n = 33), technicians (26.8%, n = 30), doctors (25.9%, n = 29), and interns (17.8%, n = 20). The study's participants were predominantly aged 20-30 years (90.17%, n = 101), with smaller proportions in the 30-40 (4.5%, n = 5), 40-50 (2.7%, n = 3), and over 50 (2.7%, n = 3) age groups. Most respondents had one to five years of experience (81.25%, n = 91), with 8% (n = 9) having 5-10 years and 10.7% (n = 12) having over 10 years (Figures 1-3). The questionnaire used a 3-point Likert scale (yes, no, and maybe) and assessed three main areas: knowledge of HBV infection, attitudes toward its prevention and vaccination, and adherence to recommended practices. The responses are tabulated and presented in Table 1.

**Table 1:** KAP questionnaire on hepatitis B infection, prevention, and vaccination among healthcare professionals The data are presented as n (%) PEP: postexposure prophylaxis; HBV: hepatitis B virus; anti-HBs: hepatitis B surface antigen; KAP: knowledge, attitude, and practice

Questions/statements	Responses, n (%)		
	Yes	No	Maybe
Knowledge questions			
Hepatitis B is a viral infection that can be acquired in a hospital setting	95 (84.8)	3 (2.67)	14 (12.5)
The mode of transmission of hepatitis B could be due to needle stick injury, perinatal transmission, sexual transmission, and blood transfusion	50 (44.6)	1 (0.89)	71 (63.3)
Vaccination for healthcare professionals can prevent the occupational transmission of hepatitis B infection	105 (93.7)	7 (6.2)	0 (0)
PEP includes immunoglobulin and vaccination for hepatitis B when the source is positive	89 (76.4)	5 (4.5)	18 (16.1)
Attitude questions			
Do you believe that instrument sterilization is important to prevent transmission?	105 (93.7)	4 (3.6)	3 (2.7)
Do you believe that wearing gloves is important to prevent transmission?	105 (93.7)	3 (2.7)	4 (3.6)
Do you recommend PEP for those who had been exposed to HBV infection where the source is unknown?	90 (80.3)	6 (5.4)	16 (14.3)
Should vaccination be compulsory for all healthcare workers?	85 (75.8)	2 (1.78)	25 (22.3)
Practice questions			
Do you practice safe needle disposal protocol in your hospital?	110 (98.2)	1 (0.9)	1 (0.9)
Three doses of vaccination should be given for hepatitis B prophylaxis (0, 1, 6)	80 (71.4)	0 (0)	42 (37.5)
Antibody titers of anti-HBs more than 10 mIU/mL can protect healthcare workers from occupational transmission of infection	92 (82.1)	9 (8.3)	11 (9.8)
Completion of the hepatitis B vaccination schedule is mandatory for all individuals who are working in a hospital/healthcare setting	110 (98.2)	1 (0.9)	1 (0.9)

Knowledge of HBV infection

The majority of respondents demonstrated a high level of knowledge about hepatitis B. A significant 84.8% (n = 95) correctly identified hepatitis B as a viral infection that can be acquired in a hospital setting, while 12.5% (n = 14) were uncertain, and only 2.67% (n = 3) disagreed. Regarding the modes of transmission, 44.6% (n = 50) correctly understood them, though a notable 63.3% (n = 71) were uncertain, and 0.89% (n = 1) disagreed. An overwhelming 93.7% (n = 105) acknowledged that vaccination for healthcare professionals can prevent occupational transmission of hepatitis B, with only 6.2% (n = 7) disagreeing. Additionally, 76.4% (n = 89) were aware that PEP includes immunoglobulin and vaccination when the source is positive, although 16.1% (n = 18) were unsure, and 4.5% (n = 5) disagreed.

Attitudes toward HBV prevention and vaccination

The respondents' attitudes toward preventive measures were predominantly positive. A significant 93.7% (n = 105) recognized the importance of instrument sterilization to prevent transmission, while 3.6% (n = 4) disagreed, and 2.7% (n = 3) were uncertain. Similarly, 93.7% (n = 105) agreed on the importance of wearing gloves, with minor dissent (2.7%, n = 3) and uncertainty (3.6%, n = 4). When asked about recommending PEP for exposure where the source is unknown, 80.3% (n = 90) supported it, though 14.3% (n = 16) were unsure, and 5.4% (n = 6) disagreed. Furthermore, 75.8% (n = 85) believed that vaccination should be compulsory for all HCWs, with 22.3% (n = 25) uncertain and 1.78% (n = 2) disagreeing.

Practices regarding HBV prevention and vaccination

The study also evaluated the participants' adherence to recommended practices. An impressive 98.2% (n = 110) reported practicing safe needle disposal protocols in their hospitals, with only 0.9% (n = 1) disagreeing and 0.9% (n = 1) uncertain. Regarding the number of doses for hepatitis B prophylaxis, 71.4% (n = 80) correctly answered, though 37.5% (n = 42) were unsure. Additionally, 82.1% (n = 92) understood that antibody titers of anti-HBs more than 10 mIU/mL could protect HCWs from occupational transmission, while 9.8% (n = 11) were uncertain, and 8.3% (n = 9) disagreed. Finally, 98.2% (n = 110) agreed that completing the hepatitis B vaccination schedule is mandatory for all individuals working in healthcare settings, with minimal disagreement (0.9%, n = 1) and uncertainty (0.9%, n = 1).

Table 2 computes the mean and standard deviation of KAPs among the respondents.

**Table 2:** Mean and standard deviation of KAPs regarding hepatitis B transmission, vaccination, and prevention among the respondents KAPs: knowledge, attitudes, and practices

Variable	Knowledge	Attitude	Practice
Mean ± standard deviation	6.40 ± 1.33	7.29 ± 1.04	7.11 ± 1.05

The data show that the participants' average scores for KAP are 6.40, 7.29, and 7.11, respectively. The standard deviations for these scores are ±1.33 for knowledge, ±1.04 for attitude, and ±1.05 for practice. This suggests that, on average, participants scored highest in attitude, followed by practice, and then knowledge. The variability in their scores across these three areas is relatively similar.

Comparison of age, designation, and years of experience and knowledge of HBV infection

An analysis of knowledge levels across demographics reveals that in the 20-30 age group, 83 out of 101 participants demonstrated good knowledge, with minimal poor knowledge representation in other age groups (p = 0.521). Doctors significantly outperformed other designations, scoring 27 out of 29 well (p = 0.04). Nurses and technicians showed higher rates of poor knowledge (42.1%), while interns had better scores. Experience did not significantly affect knowledge levels (p = 0.658); most with one to five years (75 out of 91) and over 10 years (11 out of 12) exhibited good knowledge. The findings are tabulated in Table 3.

**Table 3:** Comparison of age, designation, and years of experience and knowledge of hepatitis B infection The data are presented in n (%) The p value <0.05 is considered statistically significant ^a^The p value for designation is 0.04, which is statistically significant

Parameter	Knowledge		
	Poor, n (%)	Good, n (%)	p value
Age in years (n)			
20-30 (101)	18 (94.7)	83 (89.2)	0.521
31-40 (5)	0	5 (5.4)	
41-50 (3)	0	3 (3.2)	
>51 (3)	1 (5.3)	2 (2.7)	
Total (112)	19 (100)	93 (100)	
Designation (n)			
Doctor (29)	2 (10.5)	27 (29.0)	0.04^a^
Interns (20)	1 (5.3)	19 (20.4)	
Nurse (33)	8 (42.1)	25 (23.7)	
Technician (30)	8 (42.1)	22 (23.7)	
Total (112)	19 (100)	93 (100)	
Experience in years (n)			
1-5 (91)	16 (84.2)	75 (80.6)	0.658
6-10 (9)	2 (10.5)	7 (7.5)	
>10 (12)	1 (5.3)	11 (11.8)	
Total (112)	19 (100)	93 (100)	

Comparison of age, designation, and years of experience and attitude toward HBV prevention and vaccination

Attitude scores varied significantly by designation but not by age or experience. In the 20-30 age group, 54 out of 101 participants exhibited good attitudes, with negligible differences in other age groups (p = 0.640). Designation had a notable impact (p = 0.000); doctors showed a higher proportion of positive attitudes (23 out of 29) compared to nurses, who mostly had poor attitudes (26 out of 33). Technicians and interns displayed mixed attitude scores. Experience did not significantly affect attitudes (p = 0.346); most with one to five years (48 out of 91) and over 10 years (9 out of 12) demonstrated positive attitudes. The findings are tabulated in Table 4.

**Table 4:** Comparison of age, designation, and years of experience and attitude toward hepatitis B infection The data are presented as n (%) The p value of <0.05 is considered statistically significant ^a^The p value for designation is 0.00, which is statistically significant

Parameter	Attitude		
	Poor, n (%)	Good, n (%)	p value
Age in years (n)			
20-30 (101)	47 (94)	54 (53.5)	0.640
31-40 (5)	1 (2)	4 (80)	
41-50 (3)	1 (2)	2 (6.5)	
>51 (3)	1 (2)	2 (3.2)	
Total (112)	50 (100)	62 (100)	
Designation (n)			
Doctor (29)	6 (12)	23 (37.1)	0.000^a^
Interns (20)	7 (14)	13 (21.0)	
Nurse (33)	26 (52)	7 (11.3)	
Technician (30)	11 (22)	19 (30.6)	
Total (112)	50 (100)	62 (100)	
Experience in years (n)			
1-5 (91)	43 (86)	48 (77.4)	0.752
6-10 (9)	4 (80)	5 (8.1)	
>10 (12)	3 (6)	9 (55.4)	
Total (112)	50 (100)	62 (100)	

Comparison of age, designation, and years of experience and practice toward HBV prevention and vaccination

Practice scores varied significantly by designation but not by age or experience. In the 20-30 age group, 62 out of 101 participants showed good practices, with no significant differences in other age groups (p = 0.861). Designation had a substantial impact (p = 0.000); doctors and technicians had higher proportions of good practices (24 out of 29 and 24 out of 30, respectively) compared to nurses, who mostly exhibited poor practices (27 out of 33). Interns also demonstrated a higher percentage of good practices (16 out of 20). The experience did not significantly affect practice scores (p = 0.207); most with one to five years (54 out of 91) showed good practices. The findings are tabulated in Table 5.

**Table 5:** Comparison of age, designation, and years of experience and practice toward hepatitis B infection The data are presented as n (%) The p value of <0.05 is considered statistically significant ^a^The p value for designation is 0.00, which is statistically significant

Parameter	Practices		
	Poor, n (%)	Good, n (%)	p value
Age in years (n)			
20-30 (101)	39 (92.9)	62 (88.6)	0.861
31-40 (5)	1 (2.4)	4 (5.7)	
41-50 (3)	1 (2.4)	2 (2.9)	
>51 (3)	1 (2.4)	2 (2.9)	
Total (112)	42 (100)	70 (100)	
Designation (n)			
Doctor (29)	5 (11.9)	24 (34.3)	0.000^a^
Interns (20)	4 (9.5)	16 (22.9)	
Nurse (33)	27 (64.3)	6 (8.6)	
Technician (30)	6 (14.3)	24 (34.3)	
Total (112)	42 (100)	70 (100)	
Experience in years (n)			
1-5 (91)	37 (88.1)	54 (77.1)	0.207
6-10 (9)	1 (2.4)	8 (11.4)	
>10 (12)	4 (9.5)	8 (11.4)	
Total (112)	42 (100)	70 (100)	

## Discussion

In our study, we observed that the majority of respondents (90.1%, n = 101) were aged between 20 and 30 years, with a smaller representation in the older age brackets: 4.4% in the 30-40 age range and 2.6% in the 40-50 age range. This age distribution reflects a growing trend of younger individuals entering the healthcare workforce, predominantly as recent graduates or early-career professionals. This pattern aligns with findings from Ojara et al. [16] and Mursy and Mohamed [17], who also noted a similar demographic composition in their studies. When analyzing professional roles, nurses constituted the largest group at 29.4%, followed closely by technicians at 26.7%, doctors at 25.8%, and interns at 17.8%. This distribution is likely due to the broader recruitment and training of nurses and technicians, who are essential to the daily operations in clinical settings. Our findings are consistent with those of Tatsilong et al. [18], but they diverge from other studies where doctors were the predominant group, likely due to a specific focus on medical professionals in those studies [19,20]. Regarding work experience, most respondents (81%) had one to five years of experience in a hospital setting, reflecting the influx of new professionals into the healthcare sector. A smaller portion of respondents had over 10 years (10.7%) or 6-10 years (9.3%) of experience. This distribution mirrors Tatsilong et al.'s findings [18], which may be attributed to the rapid growth in the healthcare sector and the continuous recruitment of early-career workers to meet increasing demands. The smaller proportion of more experienced workers could be due to the natural attrition of older staff or their transition into specialized or administrative roles.

Our study also found that 74.8% of respondents had a good understanding of hepatitis B infection, a level of knowledge that aligns with previous studies conducted across various regions of India [21,22]. This consistency is likely attributed to effective public health campaigns and education programs targeting hepatitis B awareness. However, our findings contrast with other research that reported lower knowledge levels [23,24], possibly due to regional differences in educational outreach or access to healthcare information. Doctors scored significantly higher among the different professional groups, with 27 out of 29 correct answers (p = 0.04), followed by interns. This outcome is likely due to the more extensive and specialized training doctors and interns receive on infectious diseases, including hepatitis B, during their medical education. Conversely, nurses and technicians showed a higher incidence of poor knowledge, with 42.1% demonstrating inadequate understanding. This disparity may stem from differences in educational backgrounds and the specific focus of their training, which may not emphasize hepatitis B as thoroughly as medical programs. The significant association between HCW categories, education level, and knowledge of HBV transmission routes underscores the critical role of targeted education in enhancing understanding [22,25].

Attitudes toward hepatitis B vaccination and prevention were generally positive, with 85.8% of respondents demonstrating favorable views. This finding aligns with studies by Afihene et al. [25] and Tatsilong et al. [18], suggesting a broad recognition among HCWs of the importance of vaccination. The higher positive attitude observed among doctors compared to nurses and technicians (p = 0.000) may be due to their direct involvement in patient care and decision-making, which likely heightens their awareness of the need for vaccination. The discrepancy between our findings and those of Solanki et al. [26], where nurses exhibited better attitudes, could be attributed to variations in study populations or healthcare settings. Meanwhile, Garg et al. [22], who found no significant association between HCW categories and attitudes, underscores the variability of attitudes based on context and population.

Our study also revealed a high practice rate toward hepatitis B, with 87.2% of respondents demonstrating good practices. This consistency with similar studies [21,24,25] indicates that HCWs are generally diligent in following hepatitis B prevention protocols. The significant impact of professional designation on practice (p = 0.000), with doctors, interns, and technicians showing higher proportions of good practices compared to nurses, can be explained by the more direct roles these professionals play in patient care and infection control. While age and experience did not significantly impact KAP levels, the higher KAP levels among doctors can be attributed to their extensive training, greater access to up-to-date information, and leadership roles in clinical settings. Thus, our findings highlight the strong interrelationship between KAP concerning hepatitis B infection, vaccination, and prevention. The positive correlations between these elements suggest that improvements in one area can lead to enhancements in the others. The observed differences in KAP levels between doctors and other HCWs can be linked to factors such as doctors' extensive education and training, better access to resources and professional development opportunities, their direct responsibility for patient care and infection control, and their higher positions in the workplace hierarchy, which likely bolster their confidence and proactive approach to best practices.

Limitations of the study

The relatively small sample size may not represent the broader HCW population, limiting generalizability. Self-reported questionnaires could introduce bias, as respondents might overestimate their KAPs. The cross-sectional design captures data at a single point, making it difficult to infer causality or observe changes over time. Conducted in a specific region, the study may not reflect the KAP levels of HCWs in different areas. Response bias is possible, with more knowledgeable or interested participants being more likely to participate.

## Conclusions

Our study highlights significant variations in KAPs regarding hepatitis B among healthcare professionals. Most participants demonstrated a good understanding of hepatitis B, with 74.8% exhibiting high knowledge levels, particularly among doctors. Positive attitudes were observed in 85.8% of respondents, with significant differences across professional roles; doctors and interns showed more favorable attitudes than nurses and technicians. The adherence to recommended practices was strong, with 87.2% of participants following proper protocols, especially among doctors and technicians. However, there were notable disparities in KAP levels between different professional groups, highlighting the need for targeted educational interventions, particularly for nurses and technicians. The study's findings emphasize the critical role of professional training and experience in shaping HCWs' KAP related to hepatitis B. Despite these positive trends, the study is limited by its small sample size and regional focus, which may affect the generalizability of the results. Practical workshops, continuous medical education, and regular updates on hepatitis B guidelines will ensure that all HCWs are well informed about current best practices. Mentorship programs, where doctors share their expertise with other healthcare personnel, can further enhance knowledge transfer and foster a culture of continuous learning. Creating a collaborative environment and improving communication among HCWs can also enhance patient care by ensuring consistent and efficient practices.

## Appendices

Appendix 1

Knowledge of Hepatitis B Infection, Its Prevention, and Vaccination Among Healthcare Workers

1. Hepatitis B is a viral infection that can be acquired in a hospital setting

1)      Yes

2)      No

3)      Maybe

2. The mode of transmission of hepatitis B could be due to needle stick injury, perinatal transmission, sexual transmission, and blood transfusion

1)      Yes

2)      No

3)      Maybe

3. Vaccination for healthcare professionals can prevent the occupational transmission of hepatitis B infection^*^

1)      Yes

2)      No

3)      Maybe

4. Postexposure prophylaxis includes immunoglobulin and vaccination for hepatitis B when the source is positive

1)      Yes

2)      No

3)      Maybe

Attitude Toward Hepatitis B Infection, Its Prevention, and Vaccination Among Healthcare Workers

1. Do you believe that instrument sterilization is important to prevent transmission?

1)      Yes

2)      No

3)      Maybe

2. Do you believe that wearing gloves is important to prevent transmission?

1)      Yes

2)      No

3)      Maybe

3. Do you recommend postexposure prophylaxis for those who have been exposed to hepatitis B virus infection where the source is unknown?

1)      Yes

2)      No

3)      Maybe

4. Should vaccination be compulsory for all healthcare workers?

1)      Yes

2)      No

3)      Maybe

Practices Toward Hepatitis B Infection, Its Prevention, and Vaccination Among Healthcare Workers

1. Do you practice safe needle disposal protocol in your hospital?

1)      Yes

2)      No

3)      Maybe

2. Three doses of vaccination should be given for hepatitis B prophylaxis (0, 1, and 6)

1)      Yes

2)      No

3)      Maybe

3. Antibody titers of hepatitis B surface antigen of more than 10 mIU/mL   can protect healthcare workers from the occupational transmission of infection

1)      Yes

2)      No

3)      Maybe

4. Completion of the hepatitis B vaccination schedule is mandatory for all individuals who are working in a hospital/healthcare setting

1)      Yes

2)      No

3)      Maybe

Appendix 2

Scores of the knowledge, attitude, and practice questionnaire on hepatitis B infection, prevention, and vaccination among healthcare professionals

**Table 6:** Scores of knowledge, attitude, and practice questionnaire on hepatitis B infection, prevention, and vaccination among healthcare professionals The data are presented as n (%) The statistical analysis was carried out using the Kruskal-Wallis test ^a^The p value of <0.05 is considered statistically significant

Variable	Score	Profession of participants				
		Doctor, n (%), n = 29	Interns/student, n (%), n = 20	Nurse, n (%), n = 33	Technician, n (%), n = 30	Total, n (%), n = 112
Knowledge (X^2^ value: 49.512; p value: <0.001^a^)	2	0	0	1 (3)	0	1 (0.9)
	3	2 (6.9)	0	0	0	2 (1.8)
	4	0	0	3 (9.1)	4 (13.3)	7 (6.2)
	5	0	1 (5)	4 (12.1)	4 (13.3)	9 (8)
	6	4 (13.8)	11 (550)	22 (45.8)	11 (36.7)	48 (42.9)
	7	4 (13.8)	2 (10)	1 (3.0)	5 (16.7)	12 (10.7)
	8	19 (57.6)	6 (30)	2 (6.1)	6 (20)	33 (29.5)
Attitude (X^2^ value: 38.156; p value: <0.001^a^)	2	0	0	1 (3)	0	1 (0.9)
	4	0	0	1 (3)	1 (3)	2 (1.8)
	5	0	1 (5)	2 (6.1)	1 (3)	4 (3.6)
	6	2 (6.9)	4 (20)	1 (3)	3 (10)	10 (8.9)
	7	4 (13.8)	2 (10)	21 (63.6)	6 (20)	33 (29.5)
	8	23 (79.3)	13 (65)	7 (21.2)	19 (17)	62 (55.4)
Practice (X^2^ value: 50.288; p value: <0.001^a^)	4	0	1 (5)	1 (3	0	2 (1.8)
	5	0	1 (5)	1 (3)	0	2 (1.8)
	6	5 (10)	2 (10)	25 (75)	6 (20)	3 (33.9)
	7	0	4 (20)	1 (3)	4 (13.3)	9 (8)
	8	24 (82.8)	12 (60)	5 (15.2)	20 (66.7)	61 (54.5)
